# A new decomposition mechanism for metal complexes under water-oxidation conditions

**DOI:** 10.1038/s41598-019-43953-6

**Published:** 2019-05-16

**Authors:** Mohammad Mahdi Najafpour, Hadi Feizi

**Affiliations:** 1 Department of Chemistry, Institute for Advanced Studies in Basic Sciences (IASBS), Zanjan, 45137-66731 Iran; 2 Center of Climate Change and Global Warming, Institute for Advanced Studies in Basic Sciences (IASBS), Zanjan, 45137-66731 Iran; 3 Research Center for Basic Sciences & Modern Technologies (RBST), Institute for Advanced Studies in Basic Sciences (IASBS), Zanjan, 45137-66731 Iran

**Keywords:** Heterogeneous catalysis, Inorganic chemistry

## Abstract

Herein, water-oxidation reaction by cobalt(II) phthalocyanine, N,N′-bis (salicylidene) ethylenediamino cobalt(II), nickel(II) Schiff base (N,N′-bis (salicylidene)ethylenediamino nickel(II), nickel(II)) phthalocyanine-tetrasulfonate tetrasodium, manganese(II) phthalocyanine, 5,10,15,20-Tetraphenyl-21H,23H-porphine manganese(III) chloride, manganese(III) 5,10,15,20-tetra(4-pyridyl)-21H,23H-porphine chloride tetrakis(methochloride) was investigated using electrochemistry, UV-vis spectroscopy and spectroelectrochemistry. According to our results, a new decomposition pathway for these metal complexes under water-oxidation conditions was proposed. The produced metal oxide obtained by decomposition of metal complex under water -oxidation conditions not only catalyzes water-oxidation reaction but this metal oxide also accelerates decomposition of the corresponding complex to form higher amounts of the metal oxide. We hypothesize that such a mechanism could be investigated for many metal complexes under different oxidation or reduction reactions.

## Introduction

Hydrogen is a promising molecule to store sustainable, intermittent and fluctuating energies^1^. An approach to producing hydrogen is water-splitting reaction. For water-splitting reaction, efficient and stable water-oxidizing and water-reducing catalysts are necessary to be designed and synthesized^2,3^. Between water-oxidation and water-reduction reactions, water-oxidation reaction is a bottleneck for water splitting^4–10^. A wide variety of first-row transition metal complexes, such as Mn, Fe, Co, Ni, and Cu complexes, are interesting to be used for water-oxidation reaction since they are inexpensive and environmentally friendly^4–10^.

Many metal complexes with Schiff base, phthalocyanine, pyridine, porphyrin, diarylphosphinate, and other N and O donor ligands were reported as water-oxidizing catalysts under different conditions^4–10^. In the presence of these metal complexes and under water-oxidation reaction, finding a true catalyst remains a challenging issue^11–15^. Although many research groups proposed a molecular-based mechanism for water-oxidation reaction in the presence of metal complexes, a few research groups suggested an oxide-based mechanism for water-oxidation reaction in the presence of many molecular-based catalysts^11–15^. In other words, it is proposed that in some cases a metal complex is not stable and may decompose to metal oxides; subsequently, obtained metal oxides from decomposition of metal complex could serve as suitable catalysts for water-oxidation reaction. However, the details of the decomposition of metal complexes under water-oxidation reaction remain unknown.

It was reported that metal oxides in the presence of oxidants served as remarkable materials for decomposition of dye^16^. As metal oxides were reported to be a decomposition agent for organic compounds, it is needed to explore if formed metal oxides could affect the metal complexes under water-oxidation reactions. We suggested that metal oxides, not only could serve as water-oxidizing catalyst but also are able to oxidize metal complexes. Indeed, the obtained metal oxides by decomposition of metal complexes are usually nanosized particles, and thus, their activities are higher than the bulk metal oxides.

Herein, we reported a decomposition mechanism for Mn, Co and Ni-based complexes (Fig. S1) under water-oxidation reaction.

## Results

**Figure 1:**
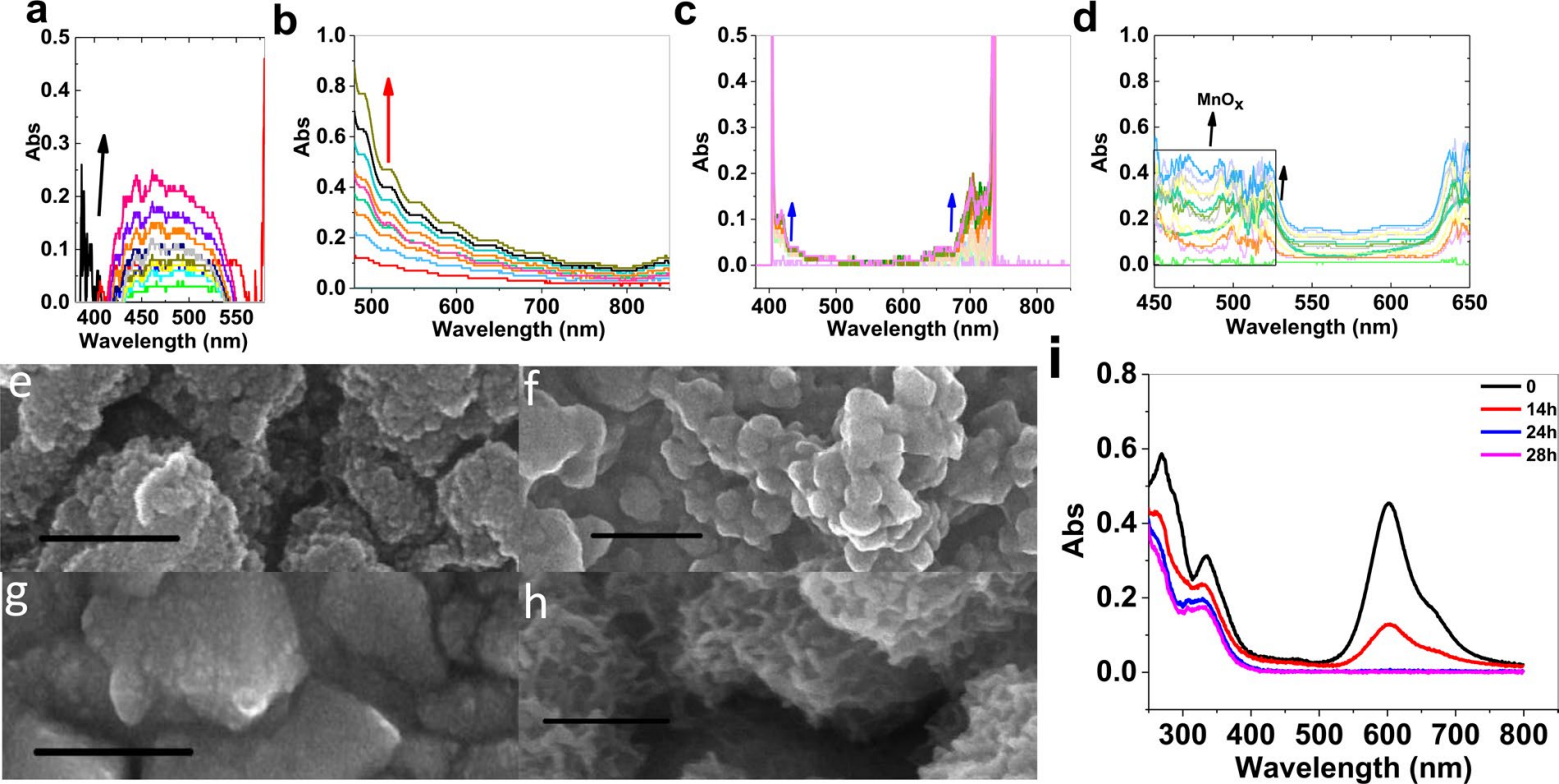
Spectroelectrochemistry in the presence of Ni phet (**a**), Ni Sch (**b**), Co Sch (**c**) and Mn phet (**d**). For the conditions see: Table S3 and Fig. S2. SEM images of FTO after the amperometry in the presence of Ni phet (**e**), Ni Sch (**f**), Co Sch (**g**) and Mn phet (**h**). For the conditions see Table S6. UV-vis spectra for Ni phet under potential. The conditions**:** in the phosphate buffer (20.0 mL; 0.25 M) at pH = 11.0 during 28 hours. The scale bars for SEM images are 200 nm. Arrows show an increase for UV-vis absorbance spectra.

Decomposition reactions of metal complexes (Fig. S1) in the presence of a fluorine-doped tin oxide electrode (FTO) under water-oxidation conditions were carried out. As shown in Fig. 1, Mn, Co and Ni complexes (Fig. S1) were slowly decomposed under water-oxidation reaction, and as previously reported, the corresponded metal oxides were formed^17–19^. Such decomposition can significantly degrade the complex (Fig. 1; the reference electrode in the paper is Ag/AgCl/KCl_sat_).

In the next step, metal complexes were investigated in the presence of the corresponding metal oxides covered on FTO in the absence of potential. The aim of this step was to track the adsorption of the metal complexes on the surface of the metal oxide covered on FTO in the absence of water-oxidation reaction. As shown in Fig. S4, the adsorption of metal complexes on the surface of the metal oxide covered on FTO is low.

**Figure 2:**
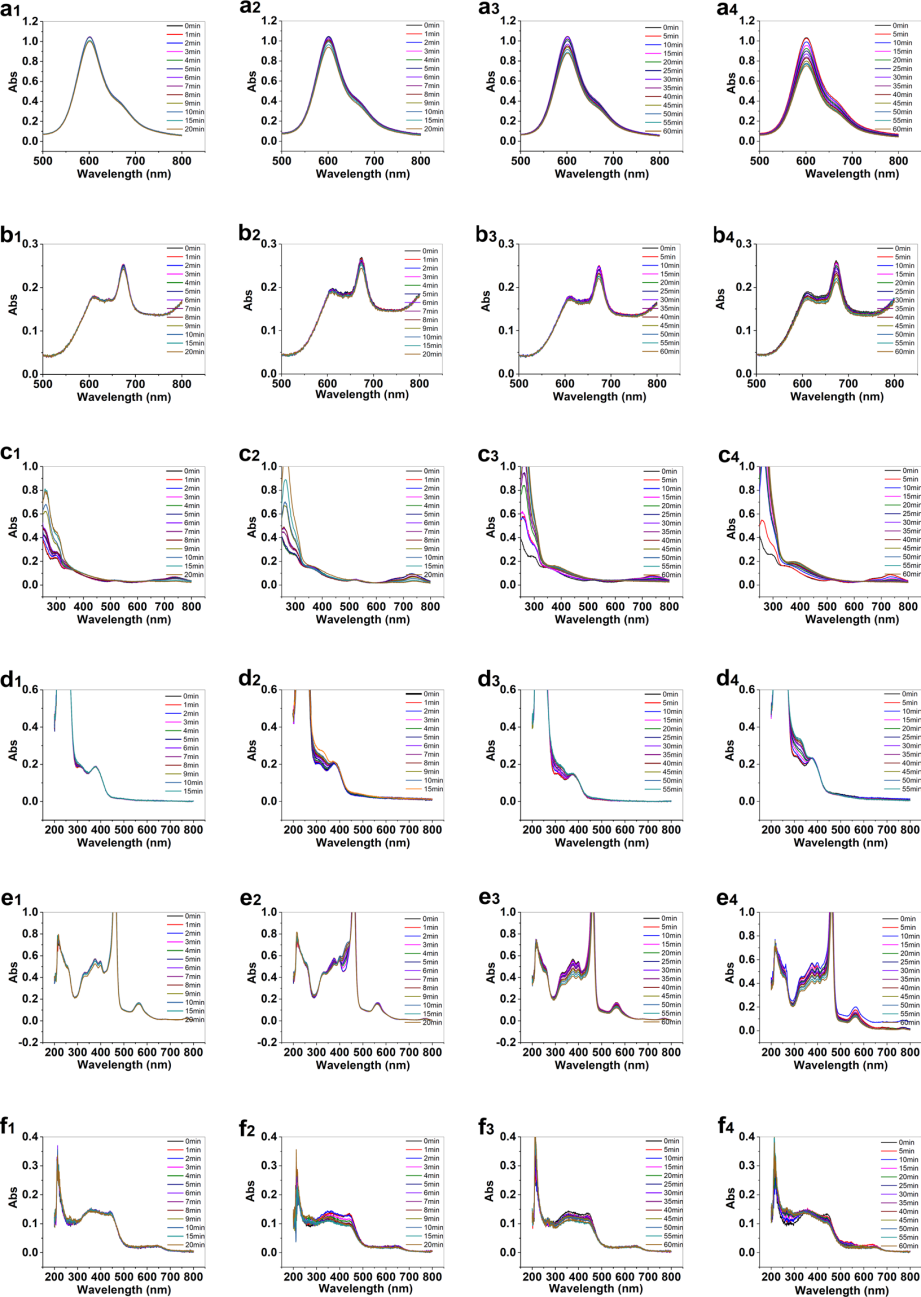
Spectroelectrochemistry in the presence of Ni phet (**a1**–**a4**; water-oxidation reaction in a short time in the presence of a bare FTO (**a1**) and the corresponded metal oxide on the FTO (**a2**); water-oxidation reaction for long time in the presence of a bare FTO (**a3**) and the corresponded metal oxide on the FTO (**a4**)), Co phet (**b1**–**b4**), Mn phet (**c1**–**c4**), Co Sch (**d1**–**d4**), Mn spor (**e1**–**e4**) and Mn por (**f1**–**f4**). The conditions: see Table S4, see also Figs S3 and S5.

Then, decomposition of the metal complexes in the presence and absence of the covered metal oxide on FTO was investigated (for the structure of complexes and set up see Fig. S1 and S2, respectively). Under water-oxidation reaction, as shown in Fig. 2, decomposition of these metal complexes is high in the presence of the corresponded metal oxide. A decrease of concentration of metal complex in solution corresponds not to the adsorption of metal complex on metal oxide (Fig. S4), but is related to the decomposition of metal complex on the surface of metal oxide. The current is also higher in the presence of metal oxides (Table S1 and Fig. S6). Indeed, in the presence of metal oxides, the relative rates for the decomposition reaction are up to seven times greater than the decomposition reaction in the absence of metal oxides (Table S2).

Based on obtained experimental data, metal oxides could accelerate the decomposition reaction of metal complexes under water-oxidation conditions. It is hypothesized that under water-oxidation reaction, metal complexes would be decomposed into metal oxides that not only could oxidize water but also have the potential of being a desired catalyst for the decomposition of the corresponded metal complexes.

**Figure 3:**
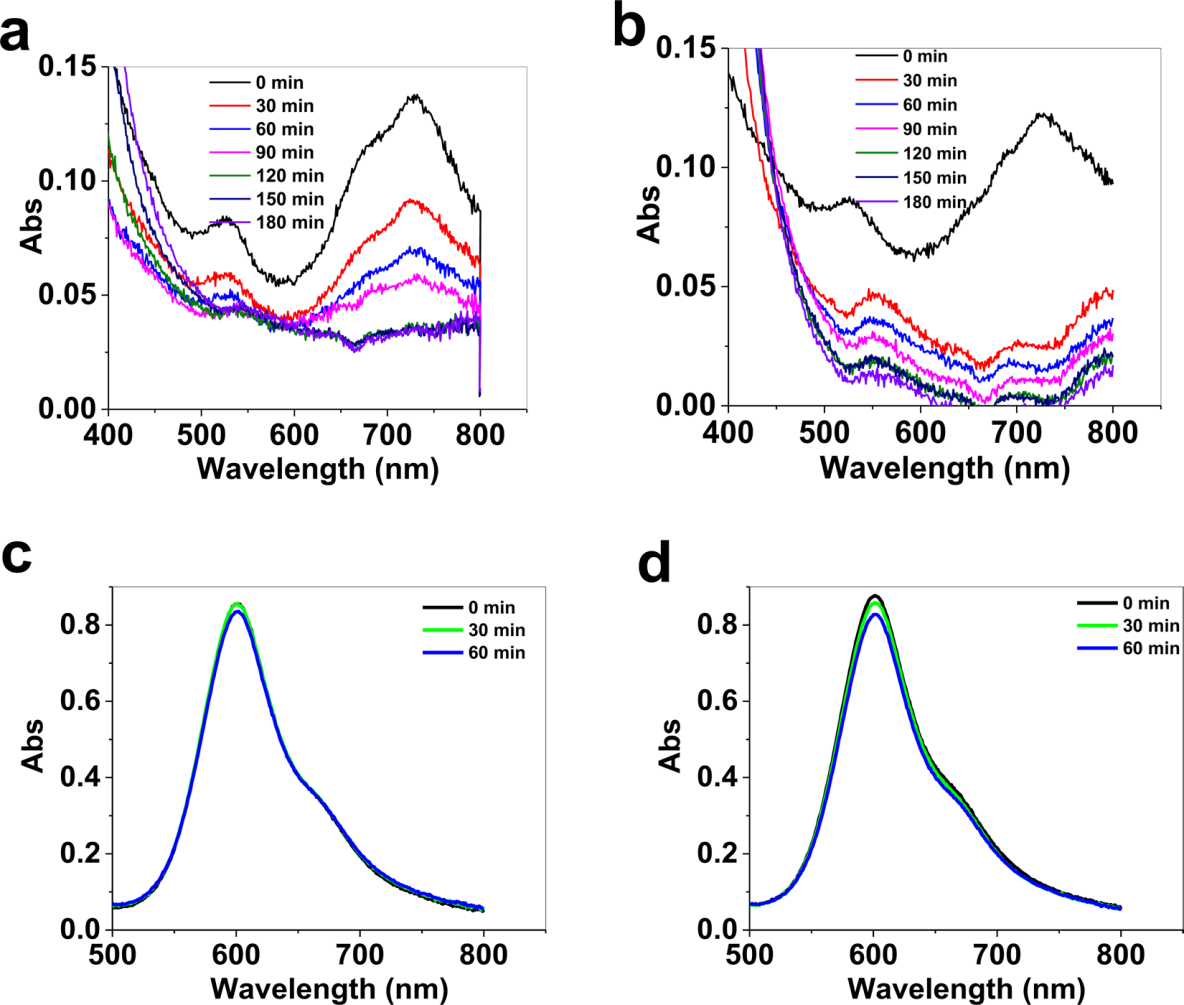
Spectroelectrochemistry under water-oxidation reaction in the presence of Mn phet in the presence of a bare FTO (**a**) and the presence of the decomposed product of the same complex on FTO (**b**). Spectroelectrochemistry under water-oxidation reaction in the presence of Ni phet in the presence of a bare FTO (**c**) and the presence of the decomposed product of the same complex on FTO (**d**). Amperometric conditions: 1.60 V. For the conditions see Table S5.

The precipitated oxides on the surface of FTO could be detected by spectroelectrochemistry (Fig. S2). Such cases for Co, Mn and Ni complexes are shown in Fig. 1e–h. These oxides are nanosized particles with different shape and morphology depends on the ligand and the conditions of reaction (Fig. 1e–h). The effect of the produced Mn oxide on the decomposition of the Mn phet was also investigated in another experiment. As shown in Fig. 3, manganese oxide covered on FTO was obtained by the decomposition of the Mn phet under water-oxidation conditions; the decomposition of Mn phet in the presence of this FTO was significantly more than a fresh FTO. This experiment showed that Mn oxide accelerates Mn phet decomposition. A similar character was observed for Ni phet (Fig. 3).

## Discussion

**Figure 4:**
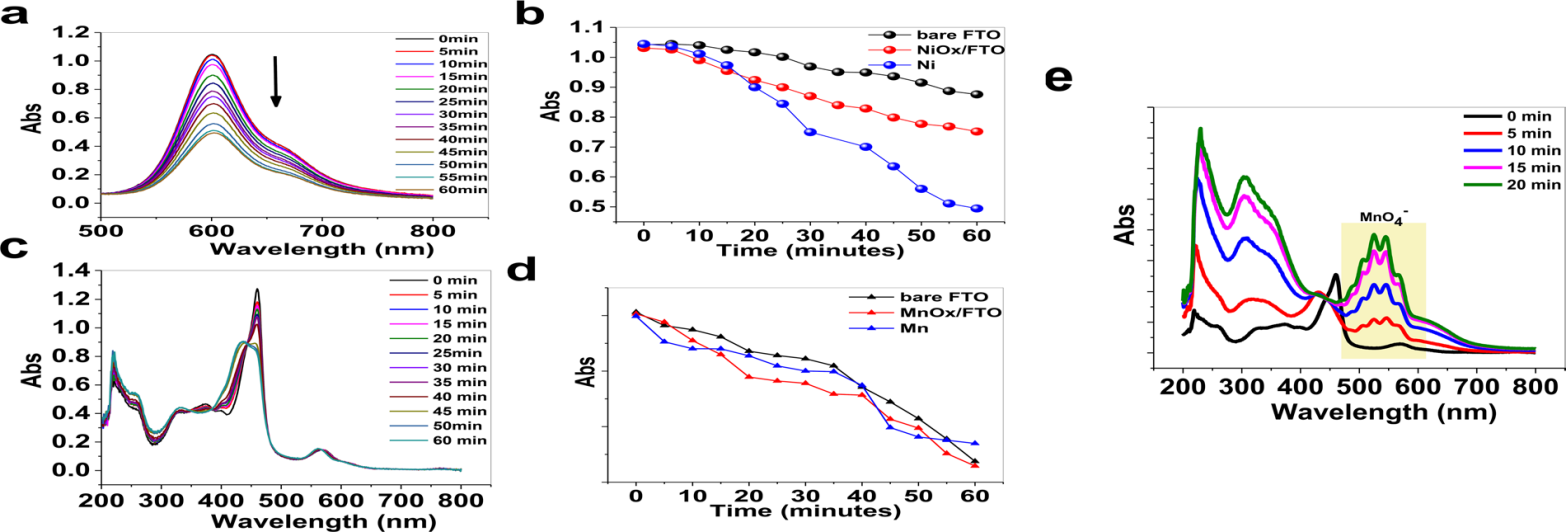
Spectroelectrochemistry in the presence of Ni phet and Ni foam (**a**). The conditions: in the phosphate buffer (5.0 mL;0.25 M) at pH = 11.0 (amperometric conditions: 1.60 V). The absorption at 600 nm for water-oxidation reaction in the presence of a bare FTO, NiO_x_/FTO and Ni foam (**b**). Spectroelectrochemistry in the presence of Mn spor and Mn chips (**c**). The conditions: in the phosphate buffer (5.0 mL; 0.25 M) at pH = 11.0 (amperometric conditions: 0.8 V). The absorption at 460 nm for water-oxidation reaction in the presence of a bare FTO, MnO_x_/FTO and Mn chips (**d**). The conditions: in the phosphate buffer (5.0 mL; 0.25 M; at pH = 11.0; amperometric conditions: for Mn chips 0.8 V and for a bare FTO and MnO_x_/FTO 1.6 V). Spectroelectrochemistry in the presence of Mn spor and Mn chips (**e**). The conditions: in the phosphate buffer (5.0 mL; 0.25 M; at pH = 11.0; amperometric conditions: for Mn chips 1.6 V.

Under water-oxidation conditions, the decomposition of metal complexes (Fig. S1) in the presence of higher load of the metal oxides showed a significant increase. For instance, Ni phet showed highly decomposition in the presence of an Ni foam. As shown in Fig. 4a, for the degradation of Ni phet by Ni foam or a bare FTO a time-delay was observed, but such a time-delay for NiO/FTO electrode is not displayed (Fig. 4b). It is hypothesized that Ni oxide formation on a bare FTO or Ni foam, which accelerates the degradation of metal complex needs time to form and, thus, a time-delay was observed. In contrast, an obtained NiO/FTO electrode immediately decomposed Ni phet.

On the other hand, the higher amounts of Ni oxides on the Ni foam at 1.6 V or Mn chips even at 0.8 V significantly increase the decomposition reaction (Fig. 4). Interestingly, at 1.6 V Mn is oxidized to MnO_4_^−^ as a strong oxidant. The oxidant decomposed the complex immediately (Fig. 4e). This experiment clearly showed that a high-valent metal oxide-based compound (e.g., MnO_4_^−^ in this case), which was produced under water-oxidation reaction, had an important role in decomposition of the metal complex.

If one of the reaction products is a catalyst for the same reaction, it is called a self-catalysis reaction. In this case, the reaction of the decomposition of a complex could be called a self-decomposition reaction because metal oxides obtained by decomposition of metal complex are also a catalyst for the decomposed reaction.

**Figure 5:**
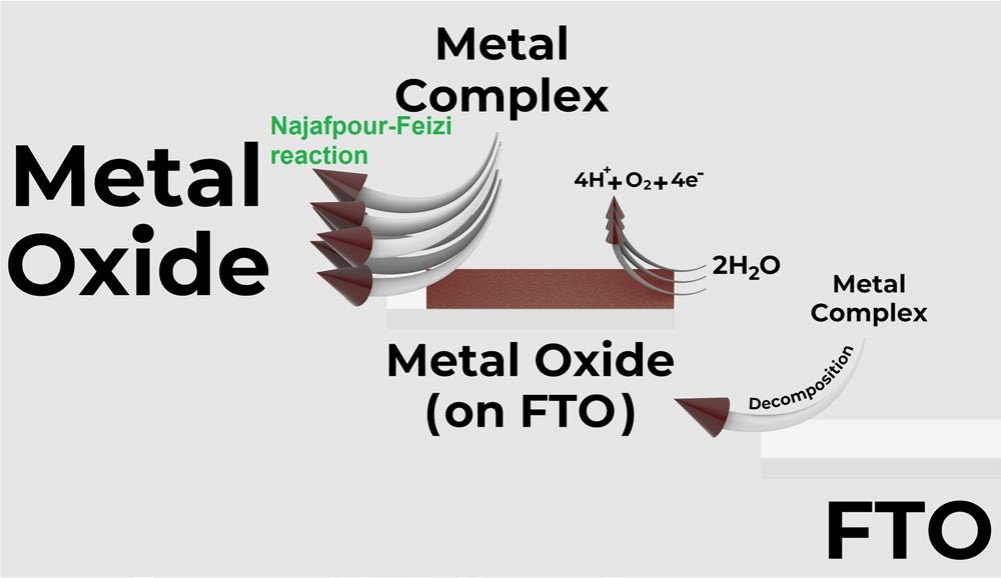
A schematic reaction to indicate Najafpour-Feizi reaction.

Our experiments showed that formed metal oxides during water-oxidation reaction not only accelerate water-oxidation reaction but also drive the decomposition of metal complexes. This self-decomposition reaction (Najafpour-Feizi reaction; Fig. 5; see also supplementary animation) is critical and could be investigated for other oxidation reactions because the reaction could indicate the details of the decomposition of the metal complexes in the presence of the metal oxide under the oxidation reaction.

## Conclusions

To find out the details of the effect of the metal oxide on the metal complexes under water-oxidation reaction, the effect of metal oxides on the decomposition of a wide variety of metal complexes under water-oxidation reaction was studied. By the results, we propose a new decomposition pathway (Najafpour-Feizi reaction) for some metal complexes under water-oxidation reaction. Under these conditions, the role of the metal oxide is not only simplify water-oxidation reactions on the surface of fluorine-doped tin oxide electrode but also accelerates the decomposition of the complex to form higher amounts of the metal oxide. We showed that such a phenomenon could occur for Mn, Co and Ni complexes under water-oxidation reaction. This phenomenon is important because it shows a few amounts of formed metal oxide under water-oxidation reaction could result in the high decomposition of the metal complex.

As a further outlook, we suggest that such a reaction may occur for other oxidation reactions such as epoxidation, alcohol or sulfide oxidation and even corresponded photochemical reactions. Equivalently, we hypothesize that for reduction reactions in the presence of metal complexes, the role of the formed metallic nanoparticles on the decomposition of metal complexes during the reaction should be investigated.

## Experimental

### Materials

All reagents were of analytical grade and were used as received without any further purification. Nickel(II) phthalocyanine-tetrasulfonate tetrasodium salt (Ni phet), manganese (II) phthalocyanine (Mn phet), nickel(II) Schiff base (*N*,*N*′-bis (salicylidene)ethylenediamino nickel(II)) (Ni Sch), N,N′-bis (salicylidene) ethylenediamino cobalt(II) (Co Sch), cobalt(II) phthalocyanine (Co phet), 5,10,15,20-Tetraphenyl-21H,23H-porphine manganese(III) chloride (Mn por), manganese(III) 5,10,15,20-tetra(4-pyridyl)-21H,23H-porphine chloride tetrakis(methochloride) (Mn spor) and fluorine-doped tin oxide coated glass (FTO) were purchased from Sigma-Aldrich. An EmStat^3+^ from PalmSens (Netherlands) was used for the electrochemical experiments. The amperometry was performed with a conventional three-electrode setup, in which FTO, Ag/AgCl/KCl_sat_ and a platinum foil served as working, reference and auxiliary electrodes, respectively. The distance between two opposite sides of the FTO was measured by a digital caliper MarCal 16ER model (Mahr, Germany). The temperature was measured by Laserliner 082 (Germany).

## Supplementary information

Related Manuscript File

supplementary information file
