# Supplementary material for: A new decomposition mechanism for metal complexes under water-oxidation conditions
Source: Sci Rep. 2019 May 16;9:7483. doi: 10.1038/s41598-019-43953-6 (PMC6522543; doi:10.1038/s41598-019-43953-6)
Supplement: Supplementary file 1 — Related Manuscript File [file 41598_2019_43953_MOESM1_ESM.doc]

**Supplementary Information**

**A new decomposition mechanism for metal complexes under water-oxidation conditions**

Mohammad Mahdi Najafpour^*1-3^ and Hadi Feizi^1^

^1^Department of Chemistry, Institute for Advanced Studies in Basic Sciences (IASBS), Zanjan, 45137-66731, Iran

^2^Center of Climate Change and Global Warming, Institute for Advanced Studies in Basic Sciences (IASBS), Zanjan, 45137-66731, Iran

^3^Research Center for Basic Sciences & Modern Technologies (RBST), Institute for Advanced Studies in Basic Sciences (IASBS), Zanjan 45137-66731, Iran

Figure S1 The structure of cobalt(II) phthalocyanine (a: Co phet), N,N′-bis (salicylidene) ethylenediamino cobalt(II) (b: Co Sch), nickel(II) Schiff base (N,N′-bis (salicylidene)ethylenediamino nickel(II)) (c: Ni Sch), nickel(II) phthalocyanine-tetrasulfonate tetrasodium (d: Ni phet), manganese(II) phthalocyanine (e: Mn phet), 5,10,15,20-Tetraphenyl-21H,23H-porphine manganese(III) chloride (f: Mn por), manganese(III) 5,10,15,20-tetra(4-pyridyl)-21H,23H-porphine chloride tetrakis(methochloride) (g: Mn spor).


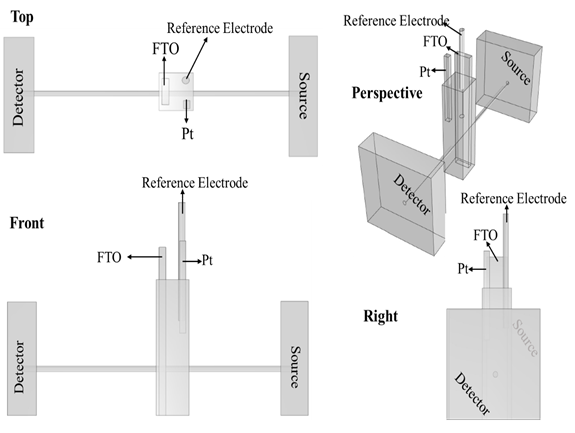


Figure S2 Schematic image for the set-up of Figure 1.


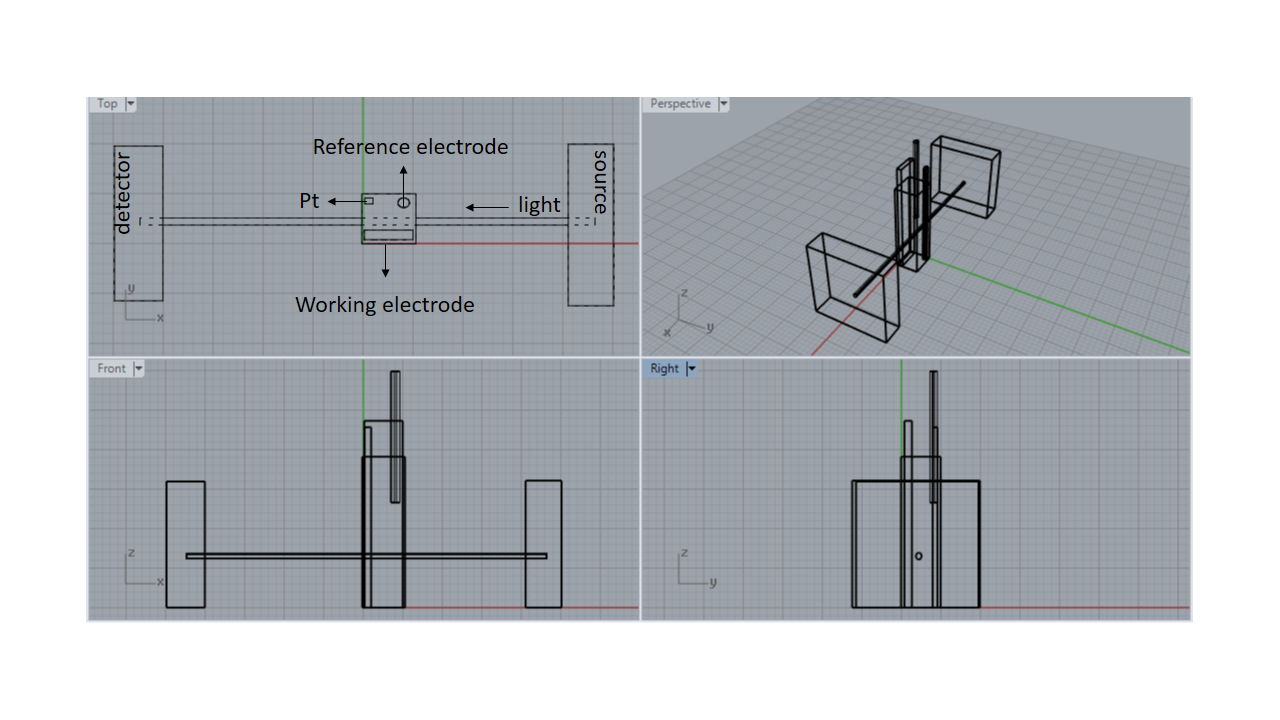


Figure S3 Schematic image for the set-up of Figure 2.

Figure S4 UV-vis spectra for the adsorption for Mn por (a), Mn spor (b), Ni phet (c), Co phet (d), Co Sch (e) on the metal oxide covered on FTO.

Figure S5 Spectroelectrochemistry in the presence of Ni Sch under water-oxidation reaction in a short time in the presence of a bare FTO (a) or the attributed metal oxide on the FTO (b). The conditions: see Table S4.

Figure S6 The current for the metal complex for 20 minutes in the absence (a) and the presence of metal oxide (b). The current for the metal complex for 60 minutes in the absence (c) and the presence of metal oxide (d).

|  | The current in the absence of metal oxide  during 20 min (µA) | The current in in the presence of metal oxide during 20 min (µA) | The current in in the absence of metal oxide  during 60 min (µA) | The current in the presence of metal oxide  during 60 min (µA) |
| --- | --- | --- | --- | --- |
| Co phet | 595.9 | 2991.0 | 407.7 | 1494.1 |
| Co Sch | 2167.0 | 3960.5 | 2031.5 | 2776.7 |
| Ni Sch | 1805.4 | 7407.5 | - | - |
| Ni phet | 1360.4 | 9985.6 | 1442.8 | 8087.7 |
| Mn phet | 208.1 | 232.5 | 49.5 | 195.3 |
| Mn spor | 1203.9 | 1530.7 | 872.7 | 782.5 |
| Mn por | 748.9 | 681.0 | 759.7 | 765.3 |

Table S1 The current in the absence or the presence of metal oxide during 20 or 60 minutes.

Table S2 The relative rate of the decomposition of the complex in the absence or presence of metal oxides.

|  | Relative rate  in the presence of metal oxide/ in the absence of metal oxide |
| --- | --- |
| Ni phet | 1.7 |
| Co phet | 1.7 |
| Mn phet | 1.22 |
| Mn spor | 1.20 |
| Mn por | 7.2 |

Table S3 The experimental conditions for the electrochemical experiments.

|  | phosphate buffer (0.25M, pH=11) | acetonitrile | DMF | Tetraethylammonium perchlorate (0.25M) | Reference electrode | operation  potential |
| --- | --- | --- | --- | --- | --- | --- |
| Ni phet (0.2 mM) | 4ml | ------- | ------ | --------- | Ag/AgCl/KCl_sat_ | 1.6V |
| Ni Sch (0.3 mM) | 3ml | 1ml | ------ | --------- | Ag/AgCl/KCl_sat_ | 1.6V |
| Co Sch (8.75 nM) | 3ml | ------- | 1ml | --------- | Ag/AgCl/KCl_sat_ | 1.5V |
| Mn phet (saturated) | ------ | 3ml | ------ | 1ml | Ag/AgCl/KCl_sat_ | 1.6V |

Table S4 The experimental conditions for the electrochemical experiments.

|  | phosphate buffer (0.25M, pH=11) | acetonitrile | Tetraethylammonium perchlorate (0.25M) | DMF | Reference electrode | operation  potential |
| --- | --- | --- | --- | --- | --- | --- |
| Ni phet (0.05 mM) | 4ml | -------- | -------- | -------- | Ag/AgCl/KCl_sat_ | 1.6V |
| Co phet (0.05 mM) | 2ml | -------- | -------- | 1ml | Ag/AgCl/KCl_sat_ | 1.6V |
| Mn phet (0.05 mM) | -------- | 0.35ml | 3ml | -------- | Ag/AgCl/KCl_sat_ | 1.6V |
| Co Sch (0.05 mM) | 5ml | 0.1ml | -------- | -------- | Ag/AgCl/KCl_sat_ | 1.6V |
| Mn por (0.05 mM) | 4ml | -------- | -------- | -------- | Ag/AgCl/KCl_sat_ | 1.6V |
| Mn spor (0.05 mM) | 4ml | 0.1ml | -------- | -------- | Ag/AgCl/KCl_sat_ | 1.6V |
| Ni Sch (0.05 mM) | 10ml | 0.1ml | -------- | -------- | Ag/AgCl/KCl_sat_ | 1.6V |

Table S5 The experimental conditions for the electrochemical experiments.

|  | phosphate buffer (0.25M, pH=11) | acetonitrile | Tetraethylammonium perchlorate (0.25M) | Reference electrode | operation  potential |
| --- | --- | --- | --- | --- | --- |
| Ni phet (0.05 mM) | 5ml | -------- | -------- | Ag/AgCl/KCl_sat_ | 1.6V |
| Mn phet (0.05 mM) | -------- | 1.35ml | 5ml | Ag/AgCl/KCl_sat_ | 1.6V |

Table S6 The experimental conditions for the electrochemical experiments.

|  | phosphate buffer (0.25M, pH=11) | acetonitrile | DMF | Tetraethylammonium perchlorate (0.25M) | Reference electrode | operation  potential |
| --- | --- | --- | --- | --- | --- | --- |
| Ni phet (0.51 mM) | 4ml | ------- | ------ | --------- | Ag/AgCl/KCl_sat_ | 1.6V |
| Ni Sch (0.3 mM) | 3ml | 1ml | ------ | --------- | Ag/AgCl/KCl_sat_ | 1.6V |
| Co Sch (8.75 nM) | 3ml | ------- | 1ml | --------- | Ag/AgCl/KCl_sat_ | 1.6V |
| Mn phet (saturated) | ----- | 3ml | ----- | 1ml | Ag/AgCl/KCl_sat_ | 1.6V |
