# Supplementary figures and images for: A new decomposition mechanism for metal complexes under water-oxidation conditions
Source: Sci Rep. 2019 May 16;9:7483. doi: 10.1038/s41598-019-43953-6 (PMC6522543; doi:10.1038/s41598-019-43953-6)

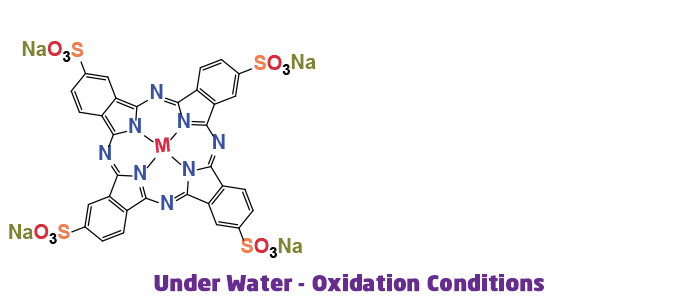

Supplement: Supplementary file 2 — supplementary information file [file 41598_2019_43953_MOESM2_ESM.gif]
